# New device for taking nine-directional ocular photographs: “9Gaze” application

**DOI:** 10.16910/jemr.15.1.5

**Published:** 2022-03-06

**Authors:** Toshiaki Goseki, Keiko Kunimi, Naoko Shioya, Yuka Iijima, Manami Sebe, Karin Hosoya, Kyo Fukaya

**Affiliations:** Atami Hospital, Shizuoka, Japan; Kitasato University, Kanagawa, Japan; The first two authors equally contributed to the first authorship

**Keywords:** eye movement, 9Gaze, nine-direction, ocular photograph, digital camera, iPad, iPod touch, gaze, eye tracking, usability

## Abstract

This study compared the time required to produce nine-directional ocular photographs using the conventional method to that using the newly devised 9Gaze application. In total, 20 healthy adults, 10 adult patients with strabismus, and 10 pediatric patients with amblyopia or strabismus had their ocular photographs taken using a digital camera with PowerPoint 2010, and with an iPad, and iPod touch with 9Gaze. Photographs of 10 healthy patients were taken by orthoptists with <1 year of experience, and the other participants had theirs taken by those with >1 year of experience. The required time was compared between the three devices in all patients and the two orthoptist groups in 20 healthy adults (>1 year and <1 year of experience). The required times were significantly different between the devices: 515.5 ± 187.0 sec with the digital camera, 117.4 ± 17.8 sec with the iPad, and 76.3 ± 14.1 sec with the iPod touch. The required time with the digital camera was significantly different between the two orthoptist groups (404.7 ± 150.8 vs. 626.3 ± 154.2 sec, *P*=0.007). The use of the 9Gaze application shortened the recording time required. Furthermore, 9Gaze can be used without considering the years of experience of the examiner.

## Introduction

In treating strabismus, the assessment of eye movements helps in the diagnosis, as well as in pre- and postoperative recordings. The simultaneous movements of both eyes in the same direction, called “versions” (10), would be disrupted if there is over- or under-functioning of the extraocular muscles and/or incomitant eye movements (1). Thus, photographs of ocular motility can assist with documentation of any improper muscle action. These images reinforce the physician's clinical impression and serve as baseline information prior to surgery. In particular, patients with strabismus and cranial nerve palsies require motility documentation. However, this is not a simple test; it requires the preparation of a jaw stand, detailed setting of the distance between the camera and the jaw stand before quantification and analysis, and time to edit the photographs after they are taken (4). Moreover, this test has the disadvantage of low reproducibility because it depends on the examiner’s skills. Although this is an important examination, it is also burdensome for patients and examiners (2). In the conventional method, after taking pictures with a digital camera, the images are imported to a personal computer (PC) and edited using Microsoft PowerPoint. This is not a simple process because editing takes a long time. The “9Gaze” application for digital devices enables the taking of photos in the nine directions of gaze (6). This application can easily create the nine-direction ocular photograph as a composite photo of nine images according to the displayed eye position. However, to the best of our knowledge, no previous study has evaluated the convenience of using 9Gaze.

This study aimed to compare the time required to produce the nine-direction ocular photograph under conventional and new methods, i.e., the digital camera and the 9Gaze application, respectively. 

## Methods

### Participants

This study was approved by the ethics committee of our hospital (21-A-186) and adhered to the tenets of the Declaration of Helsinki. Informed consent was obtained from all participants after the study details were explained.

### Materials

In total, 20 healthy adults, 10 adult patients (>20 years old) with strabismus (adult group), and 10 pediatric patients (<10 years old) with strabismus and amblyopia (pediatric group) were included in the study. Pediatric patients were only included if they had visual acuity equal to or better than 20/20 in one eye. Patients were randomly allocated to the adult and pediatric groups. We measured and compared the required times to take the nine-direction ocular photograph using three different methods: digital camera with PowerPoint, iPod touch with 9Gaze, and iPad with 9Gaze. Examinations with the three devices was performed in random order.

Five orthoptists served as the examiners. Three examiners had >1 year of experience (11 years, 6 years, and 4 years, respectively), while two had <1 year of experience. The required time for taking the photos were compared according to the years of experience of the examiners.

The 20 healthy adults were divided into two groups: 10 examined by orthoptists with >1 year of experience and the other 10 were examined by those with <1 year of experience. The adult and pediatric groups were examined by three examiners with >1 year of experience. The devices and software used were a digital camera (Cyber-shot DSC-T50: 7.4 million pixels, Canon Inc, Tokyo, Japan) with PowerPoint 2010 (Microsoft Corporation, WA, USA), and an iPad (5th generation: 241.2 mm × 185.7 mm, Apple Inc, CA, USA) or iPod touch (7th generation: 123.4 mm × 58.6 mm, Apple Inc, CA, USA) with 9Gaze (See Vision, LLC, Virginia, USA).

**Figure 1.**
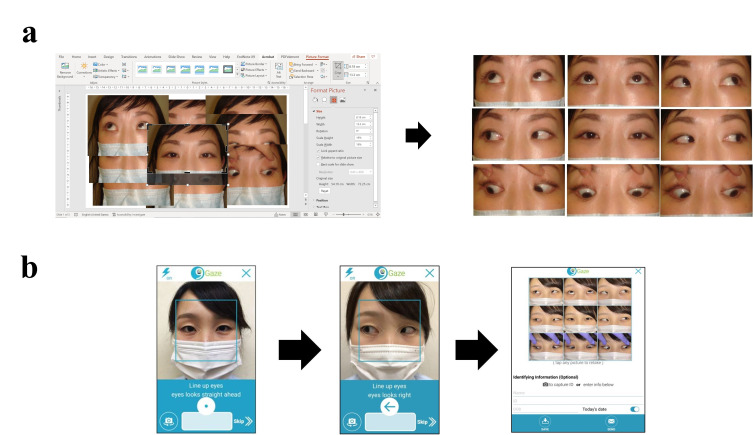
a) Details of creating the nine-direction ocular photograph with the digital camera. b) Details of creating a nine-direction ocular photograph using the 9Gaze.

### Procedure

The required time for creating a photograph with the digital camera and 9Gaze application was defined as the time from the start of the camera/application to the completion of one composite photo of the nine directions of gaze. Photographs were taken without using a chin rest to maintain the same measurement conditions. The examiner led the participants to look directly at the camera lens, and to look in the other eight directions at the tip of a pen held up, down, left, right, and diagonally (roughly 30° in each of four directions), upon which the examiner took the photographs. When the examiners took photos of the three downward directions, they always raised the participant’s eyelid manually to prevent the obstruction of the corneal margin. One examiner performed the entire procedure per patient.

The details of creating a photograph with the digital camera (conventional method) were as follows: after taking the nine directional photos, the photos were imported into the PC and pasted on a PowerPoint slide. As shown in Figure 1a, the photos were trimmed to 4.5 cm x 7.0 cm with no margins and arranged to face the nine directions in a single diagram. If a photo was not well taken, it was taken again.

The details of creating a photograph with the 9Gaze application (new method) were as follows: a picture was taken according to the eye position indicated in the shooting guide. After the nine photographs were taken, the photographs were immediately combined automatically. It was also possible to retake a photo after they had been combined (Figure 1b).

### Statistical analyses

Statistical analyses were performed using SPSS software version 27.0 (IBM, Armonk, NY, USA), and the MedCalc program for Windows version 10.4 (MedCalc Software Ltd, Ostend, Belgium). After parametric testing, the Friedman test was used to compare the required time between the three devices in the healthy participants and the adult and pediatric groups. The Mann-Whitney U test was used to compare the required time between the two examiner groups. Statistical significance was set at *p* < 0.05.

## Results

**Figure 2.**
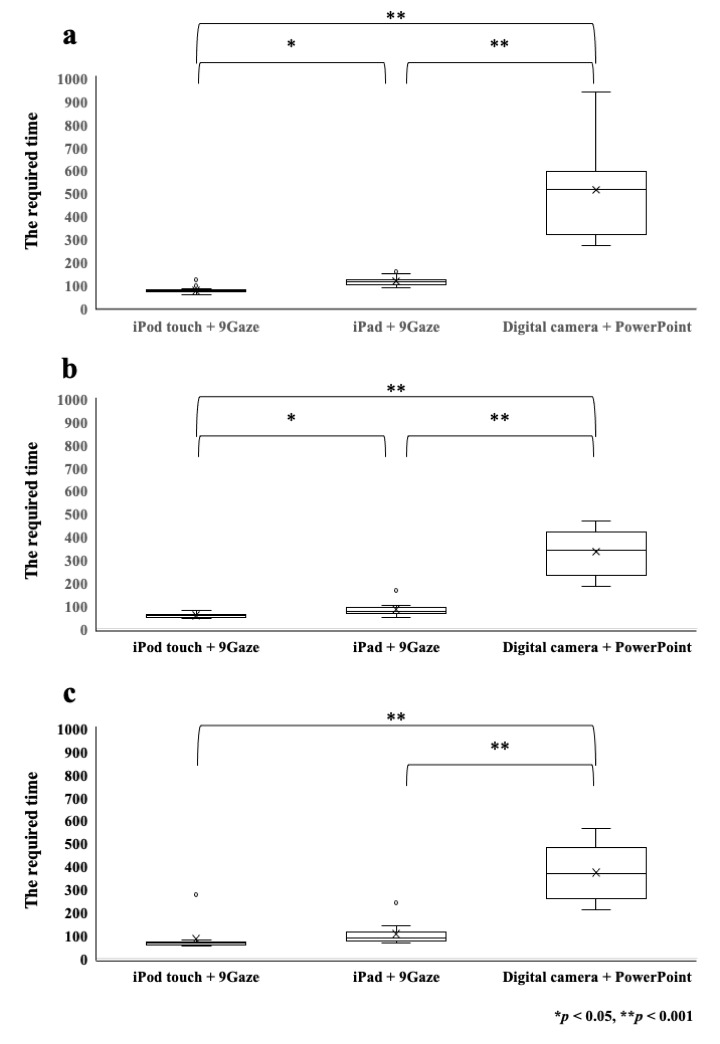
Comparison of the required times for production of the nine-directional ocular photographs between three groups: a) healthy adults, b) adult patients, c) and pediatric patients. The time required with the digital camera was significantly longer than that with the iPod touch and iPad in all groups (* p < 0.05, ** p < 0.001).

The 20 healthy adults were 26.7±4.7 (mean ± standard deviation) years old. The required time of composition of the nine-directional ocular photographs for all 20 healthy adults was 515.5±187.0 sec with the digital camera, 117.4±17.8 sec with the iPad, and 76.3±14.1 sec with the iPod touch. Significant differences were observed among the three groups, and the required time with the digital camera was significantly longer than that with the iPod touch and iPad in all groups (*p* < 0.001, Figure 2).

The adult group comprised one male and nine female. The mean age was 48.4±24.3 years. A total of four were diagnosed with sagging eye syndrome, five with exotropia, and one with esotropia. The required time for production of the nine-directional ocular photographs was 335.9±101.2 sec with the digital camera, 83.8±32.8 sec with the iPad, and 48.4±24.3 sec with the iPod touch. The pediatric group comprised six boys and four girls. The mean age was 6.9±1.9 years. A total of five of them were diagnosed with amblyopia, three with esotropia, and two with exotropia. The required times to compose the nine-directional ocular photographs were 373.9±121.8 sec with the digital camera, 106.8±51.7 sec with the iPad, and 85.1±67.9 sec with the iPod touch. The required time with the iPod touch was significantly shorter than that with the digital camera in both the adult and pediatric groups (*p* < 0.001).

The required times were 404.7±150.8 sec for the examiners with >1 years of experience and 626.3±154.2 sec for those with <1 year of experience, with the digital camera. It was followed by 117.5±21.5 sec and 117.2±14.3 sec with the iPad, and 71.9±7.5 sec and 80.7±17.9 sec with the iPod touch, respectively (Table 1). Significant difference was observed between both examiner groups only with the digital camera (*p* = 0.007: Table 1).

**Table 1.** Comparison of the time required with each device according to the years of experience of the orthoptists.

	Years of experience of the orthoptists		^a^*p* value of comparison between groups
	more than 1 year	less than 1 year	
	(mean ± standard deviation, sec)	(mean ± standard deviation, sec)	
Digital camera with PowerPoint	404.7 ± 150.8	626.3 ± 154.2	0.007
iPad with 9Gaze	117.5 ± 21.5	117.2 ± 14.3	0.85
iPod touch with 9Gaze	71.9 ± 7.5	80.7 ± 17.9	0.17

^a^ Comparison between two groups. Adjusted *p* values were analyzed by Mann-Whitney U test.

## Discussion

Although the nine-directional ocular photograph is a record, not an examination, it is helpful for preoperative and postoperative orientation, identification of paralyzed muscles, and confirmation of alphabet-type strabismus. In a previous report, analysis of the eye positions using the nine-directional eye photographs was difficult because they required detailed settings for the digital camera, chin rest, specific locations for the target, and time for editing (4).

Digital cameras have a major limitation in capturing accurate ocular photographs due to varying photograph sizes resulting from the difficulty in determining the correct distance, and the skill needed for the creation of nine-directional ocular photographs in PowerPoint. This made it difficult to compare eye movements because the size of the recordings varied before and after surgery and between examiners. On the other hand, the 9Gaze application can be used at any location. It also enables ocular photography at a consistent distance based on the shooting guide. Additionally, the time required for image processing can be shortened because there is no need to trim the images.

For example, in patients with paralytic strabismus, nine-directional ocular photographs were taken for versions and ductions for each eye. Thus, a total of twenty-seven photographs; right, left and both eyes with nine-direction ocular photographs were required. The time required for imaging was three times longer than that required for a patient with non-paralytic strabismus, resulting in a decrease in examiner productivity and an increase in patient burden. Using a digital camera would increase the time even more for the creation of the nine-directional ocular photographs, complicating such the examination of such patients. With 9Gaze, the required time was shortened to approximately 1.5 minute; this enables ophthalmologists to compose the nine-directional ocular photographs during outpatient care and use them to provide information to the patient for their informed consent for surgery. Additionally, taking images at the same magnifications enabled us to monitor patients’ state before and after surgery.

When comparing the required times for composition of the photographs between the three devices and two groups of examiners, it was shortest for the iPod touch with 9Gaze, significantly. These results suggest that the smaller device is more suited to examiners with short experience. The iPod touch is a smaller device than the digital camera and the iPad; it is easier to operate and easy to stabilize during the examination, even if the examiner is a beginner. Furthermore, the required time for composition was shortest with the iPod touch with 9Gaze, regardless of whether the patient was an adult or a child.　Based on these results, we recommend selecting a compact device equipped with 9Gaze for convenient nine-directional ocular photography. Furthermore, 9Gaze would be a more effective application if it could transmit data directly to the patient’s electronic medical records.

Upon comparing the time for examination based on the experience of the orthoptists, we observed a significant difference in the digital camera group only. The similarity in time between more and less experienced orthoptists with the 9Gaze application can be explained by its efficiency and convenience of use. It allows users to focus on taking the pictures. It is also possible to retake individual photographs even after the nine-directional ocular photographs have been compiled into a single composite photograph, without requiring examiner skill. The usefulness and simplicity of the 9Gaze application may make it a viable and recommended new method for taking nine-directional ocular photographs. As previously reported, an automatic strabismus detection system based on the Hess chart test has been developed for use in telemedicine (5; 11, 12). Incorporation of that technology with the 9Gaze application may be useful for the remote examination of patients in the future.

This study had several limitations. First, the sample size was limited, and further studies will be needed to verify our results. Second, the 9Gaze application requires that photographs are taken at about 20 cm from the participant; therefore, it is possible that the eyes’ vergence responses contained saccades that could influence the results ([Bibr b7], 8, 9) However, since the 9Gaze application is a recording method rather than a rigorous examination, we considered it useful for recording purposes irrespective of vergence responses and saccades. Previously, a novel eye-tracking device with artificial intelligence was used for nine-directional ocular measurement (3); it was trained on nine-directional ocular photographs for diagnosis of eye position abnormalities (2). Thus, it is expected that clinically simple and useful testing methods can be developed by integrating them with such devices.

## Conclusion

In conclusion, the recording of the nine-directional ocular photographs using 9Gaze shortened required time for examination. A compact and easy-to-operate device will make the 9Gaze application especially useful in clinical practice.

### Ethics and Conflict of Interest

The authors declare that the contents of the article are in agreement with the ethics described in http://biblio.unibe.ch/portale/elibrary/BOP/jemr/ethics.html and that there is no conflict of interest regarding the publication of this paper.

### Acknowledgment

We would like to acknowledge Evan Silverste, owner of See Vision, LLC (Virginia, USA), for his gracious agreement that we conduct this research and write this article. We would like to thank Editage (www.editage.com) for English language editing.
